# ﻿Phylogenetic evidence reveal a close relationship between *Amphichorda* and *Ovicillium* in Bionectriaceae (Hypocreales)

**DOI:** 10.3897/mycokeys.117.151366

**Published:** 2025-05-15

**Authors:** Yao Wang, De-Xiang Tang, Hui Chen, Qi-Rui Li, Chanhom Loinheuang, Xiang-Chun Shen

**Affiliations:** 1 The High Efficacy Application of Natural Medicinal Resources Engineering Center of Guizhou Province, Guizhou Medical University, Guian New District, Guizhou 561113, China Guizhou Medical University Guizhou China; 2 State Key Laboratory of Discovery and Utilization of Functional Components in Traditional Chinese Medicine & School of Pharmaceutical Sciences, Guizhou Medical University, Guian New District, Guizhou 561113, China National University of Laos Vientiane Laos; 3 Department of Biology, Faculty of Natural Sciences, National University of Laos, Vientiane 01080, Laos Guizhou Medical University Guiyang China

**Keywords:** Coprophilous fungi, morphology, multi-locus phylogeny, new taxa, soil fungi, taxonomy

## Abstract

Animal excrement serves as the primary substrate for *Amphichorda*, which is found in a wide range of habitats. Based on evolutionary relationships, the genus is currently classified within the Bionectriaceae. However, the phylogenetic position of *Amphichorda* and its associated taxa remains unresolved due to limited sampling in previous studies. Here, we discovered and identified five *Amphichorda* species, significantly advancing our understanding of this genus. Using six genomic loci (ITS, nrSSU, nrLSU, *tef1α*, *rpb1*, and *rpb2*) to expand taxonomic sampling, we reconstructed a phylogenetic framework for the Bionectriaceae, with a focus on *Amphichorda* and related taxa. Phylogenetic analyses revealed a close genetic connection between *Amphichorda* and related genera, yet they formed distinct clades within the Bionectriaceae and were clearly differentiated. The extensive sampling demonstrated stable phylogenetic relationships among *Amphichorda*, *Hapsidospora*, *Ovicillium*, *Proxiovicillium*, and *Bulbithecium*. Furthermore, we described two new species, *A.guizhouensis***sp. nov.** and *O.pseudoattenuatum***sp. nov.**, supported by DNA data and morphological characteristics. A comprehensive comparison of morphological traits across all members of *Amphichorda* and *Ovicillium* was conducted. This study clarifies taxonomic boundaries and evolutionary relationships within the two genera and contributes to the overall understanding of the biodiversity and systematics of the Bionectriaceae.

## ﻿Introduction

Renowned researcher E.M. Fries created the genus *Amphichorda* in 1825, designating *Amphichordafelina* (DC.) Fr. as the type species. The fungus, isolated from cat feces, was characterized by filiform conidiogenous cells and colonies with a white farinaceous hue (Fries 1825). Subsequently, Fries (1832) transferred *A.felina* to the genus *Isaria* Pers., and de Hoog (1972) formally designated *Isariafelina* (DC.) Fr. as the lectotype of the genus, following von Arx’s criteria derived from synnemata production. Based on the morphological similarity of the holoblastic conidiogenous cells, *I.felina* was accommodated in the genus *Beauveria* Vuill. as *B.felina* (DC.) J.W. Carmich. (Carmichael et al. 1980). Aside from its normal conidiogenous cells without an elongated denticulate rachis, *Amphichorda* shares a morphological resemblance with *Beauveria* (Rehner et al. 2011; Chen et al. 2013). However, phylogenetic analyses in previous studies revealed significant differences between *B.felina* and other *Beauveria* species (Hodge et al. 2005). Therefore, the genus *Amphichorda* has been reestablished with *B.felina* based on the phylogenetic relationships of internal transcribed spacer (ITS) sequences and morphology (Zhang et al. 2017). A phylogenetic analysis revealed that *Amphichorda* species were grouped in a separate clade from *Beauveria* sensu stricto, indicating the significant phylogenetic separation between the two taxa (Zhang et al. 2017). Further studies by Zhang et al. (2017, 2020) and Liu et al. (2023) placed *Amphichorda* within Cordycipitaceae and described three new species in the genus. Through a phylogenetic analysis based on a concatenated alignment of the ITS and nuclear ribosomal large subunit (nr LSU) sequences of Bionectriaceae and Cordycipitaceae, Guerra-Mateo et al. (2023) identified *Amphichorda* as a member of the Bionectriaceae family and established its close phylogenetic link to *Hapsidospora* Malloch & Cain and *Nigrosabulum* Malloch & Cain. Hou et al. (2023) determined that *Nigrosabulum* and *Hapsidospora* are congeneric, leading to their synonymization within Bionectriaceae based on both phylogeny and morphology. Leão et al. (2024) and Wang et al. (2024) also supported the placement of *Amphichorda* in the Bionectriaceae family. Despite these numerous studies, the evolutionary connections between *Amphichorda* and other genera remain unclear. It is imperative to reconstruct the phylogenetic framework for the Bionectriaceae focusing on *Amphichorda* through more sampling.

Animal excrement serves as the primary substrate for *Amphichorda*, which are found in a wide range of habitats. Currently, nine *Amphichorda* species viz. *A.cavernicola*, *A.coprophila*, *A.excrementa*, *A.felina*, *A.guana*, *A.kunmingensis*, *A.littoralis*, *A.monjolensis*, and *A.yunnanensis* have been published in reputable mycological journals (Zhang et al. 2017, 2020; Liu et al. 2023; Guerra-Mateo et al. 2023; Leão et al. 2024; Wang et al. 2024). The majority of the species are coprophilous (such as *A.coprophila*, *A.excrementa*, *A.guana*, and *A.kunmingensis*); some species are both coprophilous and entomopathogenic (such as *A.cavernicola* and *A.felina*); and one species was isolated from marine sediments (*A.littoralis*), other species from the wing surfaces of *Rhinolophusaffinis* or *Rhinolophussiamensis* (*A.yunnanensis*), or a potato dextrose agar plate partially consumed by an insect (*A.monjolensis*) (Zhang et al. 2017, 2020; Liu et al. 2023; Guerra-Mateo et al. 2023; Leão et al. 2024; Wang et al. 2024).

During an extensive mycological survey conducted in two distinct biogeographical regions spanning China and Laos, seven fungal species were isolated from diverse ecological niches, including soil substrates and animal fecal matter. To complement field-collected specimens, reference strains from the Westerdijk Fungal Biodiversity Institute’s CBS culture collection were incorporated into the study. A multilocus phylogenetic reconstruction integrating ITS, the nuclear ribosomal small subunit (nrSSU), nrLSU, the translation elongation factor 1α (*tef1α*), the largest subunit of RNA polymerase II (*rpb1*), and the second subunit of RNA polymerase II (*rpb2*) sequences confirmed their taxonomic placement within Bionectriaceae. Among these taxa, two novel species, one belonging to the genus *Amphichorda* and the other to *Ovicillium* Zare & W. Gams, were proposed and described based on morphological traits and multi-locus molecular phylogenetic data. The morphological features of every component of *Amphichorda* and *Ovicillium* were also compared in detail. In addition to introducing and characterizing these two new species, the study aimed to: (1) re-evaluate the taxonomic stability of *Amphichorda* among related genera within Bionectriaceae, (2) delineate the taxonomic boundaries and evolutionary relationships within *Amphichorda* and *Ovicillium* through comparative morphological analysis, and (3) enhance the phylogenetic resolution within Bionectriaceae using six genomic loci.

## ﻿Materials and methods

### ﻿Isolates

The samples were collected from three locations: Anshun City, China; Vientiane City, Laos; and Muang Xay District, Oudomxay Province, Laos. The techniques outlined in Wang et al. (2023) were used to isolate the strains from the soil and animal feces. Briefly, 2 g of soil was added to a flask containing 20 mL sterilized water and glass beads. The soil suspension was shaken for about 10 min and then diluted 100 times. Subsequently, 200 µL of the diluted soil suspension was spread on Petri dishes with solidified onion garlic agar (OGA: 20 g of grated garlic and 20 g of onion were boiled in 1 L of distilled water for 1 h; the boiled residue was then filtered off, and 2% (w/v) agar was added to the filtrate). Czapek yeast extract agar (CYA, Advanced Technology and Industrial Co., Ltd., China) and potato dextrose agar (PDA, Difco, USA) were used, and all media had 50 mg/L rose Bengal and 100 mg/L kanamycin added. Conidia developing on animal feces were transplanted onto plates of PDA and cultured at 25 °C. Colonies of the isolated filamentous fungi appearing in the culture were transferred onto fresh PDA media. The purified fungal strain was transferred to PDA slants and cultured at 25 °C until its hyphae spread across the entire slope. The emerging fungal spores were washed with sterile physiological saline and made into a spore suspension of 1 × 103 cells/mL. To obtain monospore cultures, a part of the spore suspension was placed on PDA using a sterile micropipette, and then a Petri dish was incubated at 25 °C. Specimens and type material were deposited in the Guizhou Medical University Herbarium (GMB), China. Living cultures were deposited at the Guizhou Medical University Culture Collection (GMBC). This investigation comprised a total of nine isolates, including species that were tentatively classified as belonging to allied genera, *Amphichorda*, and *Ovicillium* (Table 1). Cultures of four *Amphichorda* species were acquired from the CBS-KNAW Fungal Biodiversity Centre (CBS, Westerdijk Fungal Biodiversity Institute, WI, Utrecht, the Netherlands). Following transfer to PDA medium, the CBS strains were re-cultured.

**Table 1.** Species information and corresponding GenBank accession numbers of *Amphichorda* and close relative genera used in this study.

Species	Strain	Genbank accession number						Reference
		ITS	nr SSU	nr LSU	*tef1α*	*rpb1*	*rpb2*	
*Acremoniumacutatum*	CBS 682.71^T^	OQ429438	N/A	OQ055349	OQ470735	N/A	OQ453833	Hou et al. 2023
*Acremoniumalternatum*	CBS 407.66^T^	OQ429442	N/A	OQ055353	OQ470739	N/A	OQ560696	Hou et al. 2023
*Acremoniumchlamydosporium*	CBS 414.76^T^	OQ429450	N/A	OQ055361	OQ470748	N/A	OQ453844	Hou et al. 2023
Acremoniumcf.egyptiacum	CBS 270.86	OQ429463	N/A	OQ055374	OQ470760	N/A	OQ453857	Hou et al. 2023
*Acremoniumegyptiacum*	CBS 114785^T^	OQ429456	N/A	OQ055362	OQ470749	N/A	OQ453845	Hou et al. 2023
*Alloacremoniumferrugineum*	CBS 102877^T^	OQ429495	N/A	OQ055406	OQ470785	N/A	OQ453887	Hou et al. 2023
*Alloacremoniumhumicola*	CBS 613.82^T^	OQ429496	N/A	OQ055407	OQ470786	N/A	OQ453888	Hou et al. 2023
*Amphichordacavernicola*	CGMCC 3.19571^T^	MK329056	N/A	MK328961	MK335997	N/A	N/A	Zhang et al. 2020
*Amphichordacavernicola*	LC12481	MK329057	N/A	MK328962	MK335998	N/A	N/A	Zhang et al. 2020
*Amphichordacavernicola*	LC12560	MK329061	N/A	MK328966	MK336002	N/A	N/A	Zhang et al. 2020
***Amphichordacoprophila***	**CBS 173.71**	**PQ726811**	**PQ726824**	**PQ726836**	**PQ758601**	N/A	**PQ779067**	**This study**
*Amphichordacoprophila*	CBS 247.82^T^	MH861494	N/A	MH873238	OQ954487	N/A	N/A	Guerra-Mateo et al. 2023
*Amphichordacoprophila*	CBS 424.88	OQ942929	N/A	OQ943166	OQ954488	N/A	N/A	Guerra-Mateo et al. 2023
*Amphichordaexcrementa*	YFCC AECCS848^T^	N/A	OR913433	OR913439	OR917446	OR917451	OR917443	Wang et al. 2024
***Amphichordaexcrementa***	**CBS 110.08**	**PQ726812**	**PQ726825**	**PQ726837**	**PQ758602**	**PQ758614**	**PQ779068**	**This study**
***Amphichordafeline***	**CBS 250.34**	**PQ726813**	**PQ726826**	**PQ726838**	**PQ758603**	**PQ758615**	**PQ779069**	**This study**
*Amphichordafeline*	CBS 648.66	OQ942930	N/A	MH870575	OQ954491	N/A	N/A	Guerra-Mateo et al. 2023
*Amphichordaguana*	CGMCC3.17908^T^	KU746665	KY883262	KU746711	KX855211	KY883202	KY883228	Zhang et al. 2017
*Amphichordaguana*	CGMCC3.17909	KU746666	KY883263	KU746712	KX855212	KY883203	N/A	Zhang et al. 2017
***Amphichordaguizhouensis***	**GMBC 3005^T^**	**PQ726815**	**PQ726828**	**PQ726840**	**PQ758605**	**PQ758617**	**PQ779071**	**This study**
***Amphichordaguizhouensis***	**GMBC 3006**	**PQ726816**	**PQ726829**	**PQ726841**	**PQ758606**	**PQ758618**	**PQ779072**	**This study**
*Amphichordakunmingensis*	YFCC AKYYH8414^T^	N/A	OR913435	OR913438	OR917448	OR917452	N/A	Wang et al. 2024
***Amphichordakunmingensis***	**CBS 312.50**	**PQ726814**	**PQ726827**	**PQ726839**	**PQ758604**	**PQ758616**	**PQ779070**	**This study**
*Amphichordalittoralis*	FMR 17952	OQ942925	N/A	OQ943162	OQ954483	N/A	N/A	Guerra-Mateo et al. 2023
*Amphichordalittoralis*	FMR 19404^T^	OQ942924	N/A	OQ943161	OQ954482	N/A	N/A	Guerra-Mateo et al. 2023
*Amphichordalittoralis*	FMR 19611	OQ942926	N/A	OQ943163	OQ954484	N/A	N/A	Guerra-Mateo et al. 2023
*Amphichordamonjolensis*	COAD 3124^T^	OQ288256	N/A	OQ288260	OR454090	N/A	OQ405040	Leão et al. 2024
*Amphichordamonjolensis*	COAD 3125	OQ288257	N/A	N/A	N/A	N/A	OQ405041	Leão et al. 2024
*Amphichordamonjolensis*	COAD 3120	OQ288258	N/A	N/A	N/A	N/A	OQ405042	Leão et al. 2024
*Amphichordayunnanensis*	KUMCC 21-0414	ON426823	N/A	N/A	OR025977	OR022016	OR022041	Liu et al. 2023
*Amphichordayunnanensis*	KUMCC 21-0415	ON426824	N/A	N/A	OR025976	OR022015	OR022040	Liu et al. 2023
*Amphichordayunnanensis*	KUMCC 21-0416^T^	ON426825	N/A	N/A	OR025975	OR022014	OR022039	Liu et al. 2023
*Bulbitheciumammophilae*	CBS 178.78^T^	OQ429504	N/A	OQ055415	OQ470793	N/A	OQ453895	Hou et al. 2023
*Bulbitheciumarxii*	CBS 737.84^T^	OQ429505	N/A	OQ055416	OQ470794	N/A	OQ451834	Hou et al. 2023
*Bulbitheciumborodinense*	CBS 101148^T^	OQ429506	N/A	OQ055417	OQ470795	N/A		Hou et al. 2023
*Bulbitheciumellipsoideum*	CBS 993.69^T^	OQ429507	N/A	OQ055418	OQ470796	N/A	OQ453896	Hou et al. 2023
*Bulbitheciumhyalosporum*	CBS 318.91^T^	OQ429508	AF096172	OQ055419	OQ470797	N/A	OQ453897	Hou et al. 2023
*Bulbitheciumpinkertoniae*	CBS 157.70^T^	OQ429509	HQ232202	OQ055420	OQ470799	N/A	OQ453898	Hou et al. 2023
*Bulbitheciumspinosum*	CBS 136.33^T^	OQ429512	HQ232210	OQ055423	OQ470802	N/A	OQ453899	Hou et al. 2023
*Bulbitheciumtruncatum*	CBS 113718^T^	OQ429513	N/A	OQ055424	OQ470803	N/A	OQ453900	Hou et al. 2023
*Clavicepspaspali*	ATCC 13892	JN049818	U32401	U47826	DQ522321	DQ522367	DQ522416	Spatafora et al. 2007
*Clavicepspurpurea*	SA cp11	N/A	EF469122	EF469075	EF469058	EF469087	EF469105	Sung et al. 2007
***Clonostachyskunmingensis***	**GMBC 3002**	**PQ726821**	**PQ726833**	**PQ726846**	**PQ758611**	N/A	**PQ758622**	**This study**
***Clonostachysrosea***	**GMBC 3003**	**PQ726822**	**PQ726834**	**PQ726847**	**PQ758612**	**PQ779065**	**PQ779076**	**This study**
***Clonostachyssolani***	**GMBC 3004**	**PQ726823**	**PQ726835**	**PQ726848**	**PQ758613**	**PQ779066**	**PQ779077**	**This study**
*Geosmithialavendula*	CBS 344.48^T^	OQ429598	N/A	OQ055508	OQ470908	N/A	OQ453997	Hou et al. 2023
*Geosmithiapallidum*	CBS 260.33^T^	OQ429599	N/A	OQ055509	OQ470909	N/A	OQ453998	Hou et al. 2023
*Hapsidosporachrysogena*	CBS 144.62^T^	OQ429645	HQ232187	OQ055551	OQ470953	N/A	OQ454043	Hou et al. 2023
*Hapsidosporaflava*	CBS 596.70^T^	OQ429649	HQ232191	OQ055555	OQ470957	N/A	OQ454047	Hou et al. 2023
*Hapsidosporaglobosa*	CBS 512.70^T^	OQ429655	N/A	OQ055561	OQ470963	N/A	OQ454053	Hou et al. 2023
*Hapsidosporainversa*	CBS 517.70^T^	OQ429659	N/A	OQ055565	OQ470967	N/A	OQ454057	Hou et al. 2023
*Hapsidosporairregularis*	CBS 510.70^T^	OQ429660	N/A	OQ055566	OQ470968	N/A	OQ454058	Hou et al. 2023
*Hapsidosporastercoraria*	CBS 516.70^T^	OQ429662	N/A	OQ055568	OQ470970	N/A	OQ454060	Hou et al. 2023
*Hapsidosporavariabilis*	CBS 100549^T^	OQ429663	N/A	OQ055569	OQ470971	N/A	OQ454061	Hou et al. 2023
*Ovicilliumasperulatum*	CBS 426.95	KU382192	N/A	KU382233	OQ471081	N/A	OQ454166	Hou et al. 2023
*Ovicilliumasperulatum*	CBS 130362^T^	OQ429756	N/A	OQ055655	OQ471082	N/A	OQ454167	Hou et al. 2023
*Ovicilliumattenuatum*	CBS 399.86^T^	OQ429757	N/A	OQ055656	OQ471083	N/A	OQ454168	Hou et al. 2023
*Ovicilliumoosporum*	CBS 110151^T^	OQ429758	N/A	OQ055657	OQ471084	N/A	OQ454169	Hou et al. 2023
***Ovicilliumpseudoattenuatum***	**GMBC 3007^T^**	**PQ726817**	**PQ726830**	**PQ726842**	**PQ758607**	**PQ779063**	**PQ779073**	**This study**
***Ovicilliumpseudoattenuatum***	**GMBC 3008**	**PQ726818**	**PQ726831**	**PQ726843**	**PQ758608**	**PQ779064**	**PQ779074**	**This study**
*Ovicilliumsinense*	SD09701^T^	PP836762	N/A	PP836764	PP852887	N/A	N/A	Chen et al. 2024
*Ovicilliumsinense*	SD09702	PP836763	N/A	PP836765	PP852888	N/A	N/A	Chen et al. 2024
*Ovicilliumsubglobosum*	CBS 101963^T^	OQ429759	N/A	OQ055658	OQ471085	N/A	OQ454170	Hou et al. 2023
*Ovicilliumvariecolor*	CBS 130360^T^	OQ429760	N/A	OQ055659	OQ471086	N/A	OQ454171	Hou et al. 2023
*Proxiovicilliumblochii*	CBS 324.33	OQ429815	N/A	OQ430078	OQ471143	N/A	OQ454212	Hou et al. 2023
*Proxiovicilliumblochii*	CBS 427.93^T^	OQ429816	HQ232182	OQ430079	OQ471144	N/A	OQ454213	Hou et al. 2023
*Proxiovicilliumlepidopterorum*	CBS 101239^T^	OQ429817	N/A	OQ430080	OQ471145	N/A	OQ454214	Hou et al. 2023
*Proliferophialisapiculata*	CBS 303.64^T^	OQ429796	N/A	OQ055692	OQ471122	N/A	OQ454207	Hou et al. 2023
*Proliferophialisapiculata*	CBS 397.78	OQ429798	N/A	OQ055694	OQ471124	N/A	OQ454209	Hou et al. 2023
*Proliferophialisapiculata*	CBS 542.79	OQ429799	N/A	OQ055695	OQ471125	N/A	OQ454210	Hou et al. 2023
***Sesquicilliumbuxi***	**GMBC 3000**	**PQ726819**	N/A	**PQ726844**	**PQ758609**	**PQ758619**	**PQ758621**	**This study**
***Sesquicilliumcandelabrum***	**GMBC 3001**	**PQ726820**	**PQ726832**	**PQ726845**	**PQ758610**	**PQ758620**	**PQ779075**	**This study**
*Stilbocreacolubrensis*	CBS 141857^T^	MN497406	N/A	MN497409	N/A	N/A	N/A	Lechat and Fournier 2019
*Stilbocreamacrostoma*	CBS 141849	OQ429874	N/A	OQ430123	OQ471206	N/A	OQ454273	Hou et al. 2023
*Stilbocreawalteri*	CBS 144627^T^	OR050519	N/A	OQ430124	MH562714	N/A	MH577042	Voglmayr and Jaklitsch 2019
*Waltergamsiamoroccensis*	CBS 512.82^T^	OQ429943	N/A	OQ430193	OQ471276	N/A	OQ454343	Hou et al. 2023
*Waltergamsiaparva*	CBS 381.70A^T^	OQ429946	N/A	OQ430196	OQ471279	N/A	OQ454346	Hou et al. 2023
*Waltergamsiapilosa*	CBS 124.70^T^	OQ429949	N/A	OQ430199	OQ471282	N/A	OQ454349	Hou et al. 2023

### ﻿Morphological observations

After seven days (for *Ovicillium* species) or thirty days (for *Amphichorda* species) in an incubator set at 25 °C, colonies on potato dextrose agar (PDA) were macroscopically described. Characteristics and colony diameters were measured after 7 or 30 days. To take pictures of the colony characters (upper surface and reverse), a Canon 750 D camera (Canon Inc., Tokyo, Japan) was used. Colonies with micromorphological characteristics were seen at 25 °C on PDA. A light microscope (Olympus BX53) was used to examine the micro-morphological features (Conidiophores, Phialides, and Conidia) using sterile water or clear lactophenol cotton blue solution as the mounting medium.

### ﻿DNA extraction, amplification and sequencing

Axenic cultures grown on PDA plates for 14 days were used for DNA extraction. The CTAB approach, as outlined by Liu et al. (2001), was used to extract genomic DNA. The ITS, nrSSU, nrLSU, *tef1α*, *rpb1*, and *rpb2* were amplified. For the PCR amplification of six genes, the following primer pairs were employed: The ITS region was amplified using the primer combination ITS5/ITS4 (White et al. 1990). The primer pairs NS1/NS4 and LR0R/LR5 were amplified for the nrSSU region and the nrLSU region (Vilgalys and Hester 1990; White et al. 1990; Hopple 1994); the primer pairs 2218R/983F, RPB1‐5′F/RPB1‐5′R and RPB2-5′F/RPB2-5′R were amplified for the *tef1α* region, the *rpb1* region and the *rpb2* region (Rehner and Buckley 2005; Bischoff et al. 2006; Sung et al. 2007). The polymerase chain reaction (PCR) matrix was conducted in a final volume of 50 µl and all detailed information (volume, procedures, etc.) was described by Wang et al. (2023). The consensus sequences were generated by aligning forward and reverse sequencing reads with Geneious Prime 2022 (Biomatters Inc., New Zealand).

### ﻿Phylogenetic analyses

GenBank provided newly generated sequencing data (http://blast.ncbi.nlm.nih.gov/ (accessed on 20 February 2025)). The sequences mainly referred to recent articles, such as Zhang et al. (2020), Guerra-Mateo et al. (2023) and Hou et al. (2023). MAFFT v. 7 (https://mafft.cbrc.jp/alignment/server/ (accessed on 20 February 2025)) was used to create the sequence alignments for the six distinct loci (ITS, nrSSU, nrLSU, *tef1α*, *rpb1*, and *rpb2*). Where required, the aligned sequences were subsequently manually adjusted. The concatenated alignments (nrSSU + ITS + nrLSU + *tef1α* + *rpb1* + *rpb2*) were subjected to phylogenetic inferences using the maximum-likelihood (ML) and the Bayesian inference (BI) methods. IQ-tree v.2.1.3 and RAxML7.0.3 were used to conduct ML analyses using 1000 bootstrap replicates and the default general time reversible (GTR) substitution matrix (Stamatakis et al. 2008; Minh et al. 2020). ModelFinder was used to estimate the best evolutionary model for machine learning analyses (Kalyaanamoorthy et al. 2017). The TN+F+I+G4 model was selected as the optimal model for the ML analyses, with 5000 ultrafast bootstraps in a single run (Hoang et al. 2017). jModeltest v. 2.1.4 was used to estimate the optimal substitution model for Bayesian analysis and then performed using MrBayes v. 3.2.6 to assess posterior probabilities (PP) by Markov Chain Monte Carlo sampling (MCMC). For all loci in Bayesian analysis, the GTR+I+G model was suggested based on the results of jModeltest (Darriba et al. 2012; Ronquist et al. 2012). For 3,000,000 generations, four Markov chains ran simultaneously, sampling trees every 1000^th^ generations or until the average standard deviation of split frequencies fell below 0.01 to trigger an automated stop. Following the first 25% of trees being removed as part of the burn-in phase, the posterior probabilities (PP) were computed from the remaining trees. The generated trees were annotated in Microsoft PowerPoint and displayed in FigTree v. 1.4.2 (http://tree.bio.ed.ac.uk/software/figtree (accessed on 20 February 2025)). Each node of the tree displays the ML bootstrap support values (BS) greater than or equal to 80% and the relevant Bayesian posterior probability (PP) more than or equal to 0.90.

## ﻿Results

### ﻿Molecular phylogeny

**Figure 1.**
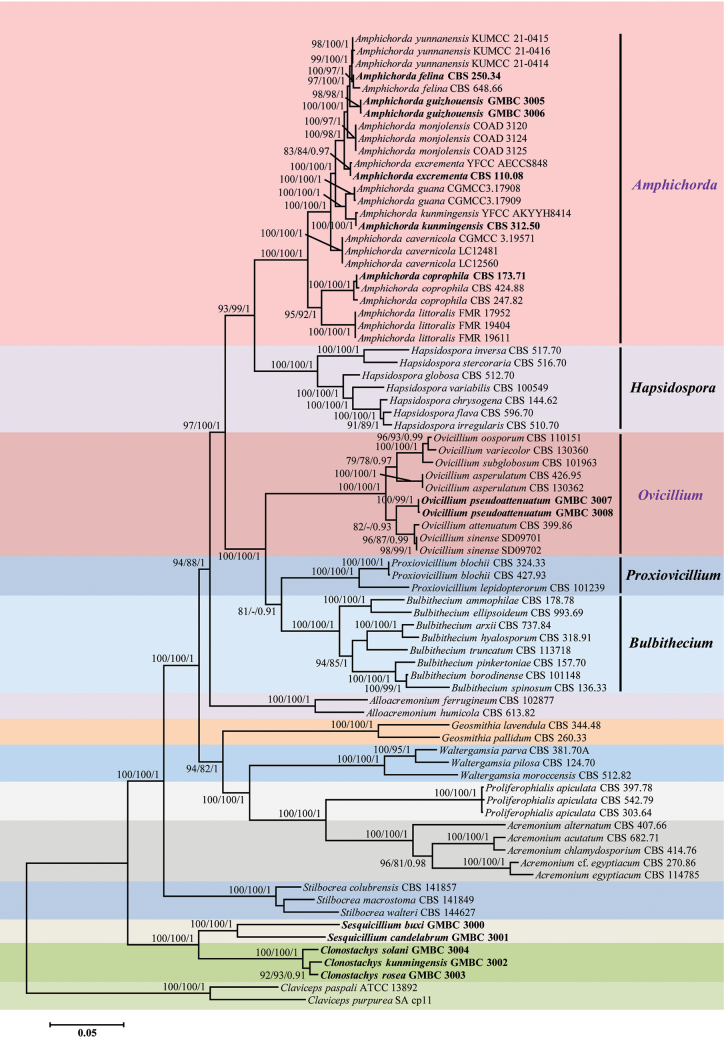
Phylogenetic relationships of *Amphichorda* and related genera in the Bionectriaceae based on combined partial nrSSU + ITS + nrLSU + *tef1α* + *rpb1* + *rpb2* sequences. Numbers at the nodes are presented here with ML bootstrap support values (BS) (IQ-TREE-BS_IQ_ > 80%/RAxML-BS_RAx_ > 80%) and relevant Bayesian posterior probabilities (PP) (PP > 0.90). Strains in bold type are those analyzed in this study.

Relevant sequences of 65 strains from GenBank were used in the phylogenetic analyses. The aim was to estimate the phylogeny of *Amphichorda* and its closely related taxa within the Bionectriaceae family. *Clavicepspaspali* (ATCC 13892) and *Clavicepspurpurea* (SA cp11) were designated as outgroup taxa for the analyses. Six concatenated loci (nrSSU + ITS + nrLSU + *tef1α* + *rpb1* + *rpb2*) were utilized to analyze the aligned DNA sequence data of 78 strains, as presented in Table 1. The final dataset consisted of 6,509 bp of sequence data, including gaps (nrSSU, 2,012 bp; ITS, 705 bp; nrLSU, 906 bp; *tef1α*, 995 bp; *rpb1*, 756 bp; and *rpb2*, 1,135 bp). Overall, the maximum likelihood (ML) trees generated by IQ-TREE and RAxML exhibited the same clades and tree structure as those obtained from the Bayesian phylogenetic analysis. With the bootstrap support values of the ML analysis (IQ-TREE-BS/RAxML-BS) and pertinent Bayesian posterior probabilities (PP) displayed at the nodes (Fig. 1), the optimal IQ-TREE tree based on the combined dataset was displayed here.

Fourteen well-supported clades were recognized based on both ML and BI analyses of the 78 taxa from Bionectriaceae and *Claviceps* (Clavicipitaceae, Hypocreales) that accommodate species of the genera *Acremonium*, *Alloacremonium*, *Amphichorda*, *Bulbithecium*, *Clonostachys*, *Geosmithia*, *Hapsidospora*, *Ovicillium*, *Proxiovicillium*, *Proliferophialis*, *Sesquicillium*, *Stilbocrea*, *Waltergamsia*, and *Claviceps* (Fig. 1). The phylogenetic analyses clearly indicated that *Amphichorda* was a monophyletic group, suggesting a common evolutionary origin. The genus *Amphichorda* had a close genetic relationship with *Hapsidospora* and *Ovicillium*, but they were clearly distinguished from their allied genera by forming three separate clades within the Bionectriaceae family. In the phylogenetic tree, the genera *Proxiovicillium* and *Bulbithecium* were also closely related to *Amphichorda*.

In this study, 13 fungal isolates were examined, including four CBS strains. The results showed that these isolates represented nine known species and two new species. The phylogenetic positions of the nine known species were evaluated according to phylogenetic inferences based on the six loci, including *Amphichordacoprophila*, *A.excrementa*, *A.felina*, *A.kunmingensis*, *Clonostachyskunmingensis*, *C.rosea*, *C.solani*, *Sesquicilliumbuxi*, and *S.candelabrum* (see Table 1, Fig. 1). The phylogenetic analyses also resolved most *Amphichorda* and *Ovicillium* lineages in separate terminal branches. It was proposed that two strains, GMBC 3005 and GMBC 3006, which formed a distinct lineage and had a close relationship with *A.felina* and *A.yunnanensis*, might be a new species in the genus *Amphichorda*, named *A.guizhouensis*. Our analyses further revealed that the newly discovered species, *O.pseudoattenuatum* (GMBC 3007 and GMBC 3008), were phylogenetically clustered with *O.attenuatum* and *O.sinense*, but it was clearly distinguished from the latter two species by forming a well-supported clade in the genus *Ovicillium* (BS_IQ_/BS_RAx_/PP = 82%/-/0.93; Fig. 1).

### ﻿Taxonomy

#### ﻿ Amphichorda guizhouensis

Taxon classificationFungiHypocrealesCordycipitaceae

Y. Wang & D.X. Tang sp. nov.

B7065AE4-BFA8-5B82-9C49-ED9650915019

857725

Fig. 2

##### Etymology.

Named after the location Guizhou Province where the species was collected.

##### Type.

China • Guizhou Province, Anshun city, Xixiu District, Liuguan Village (26.25°N, 106.22°E, 1273 m above sea level), on bird feces, 12 July 2023, Yao Wang (holotype, GMB 3005); ex-type culture, GMBC 3005.

##### Description.

**Sexual morph**: Undetermined. **Asexual morph: *Synnemata*** arising from bird feces, 1.6–2.0 mm long. Colonies on PDA attaining a diameter of 40–42 mm after a month at 25 °C, white to pinkish, flat, margin entire, reverse yellowish. ***Hyphae*** branched, smooth-walled, septate, hyaline, 0.8–2.2 μm wide. ***Conidiophores*** arising laterally from hyphae, cylindrical, straight or slightly curved, occasionally branched, hyaline. ***Conidiogenous cells*** arising laterally from aerial hyphae, basal portion cylindrical or flask-shaped, erect or irregularly curved, tapering abruptly towards the apex, 6.0–20.8 × 1.8–3.7 (X̄ = 15.2 × 2.6, n = 30) μm. ***Conidia*** 2.6–4.0 × 1.8–2.6 (X̄ = 3.1 × 2.2, n = 50) μm, one-celled, smooth-walled, hyaline, subglobose to ellipsoidal, single, often remaining attached to the apex of conidiogenous cells. ***Chlamydospores*** not observed.

**Figure 2.**
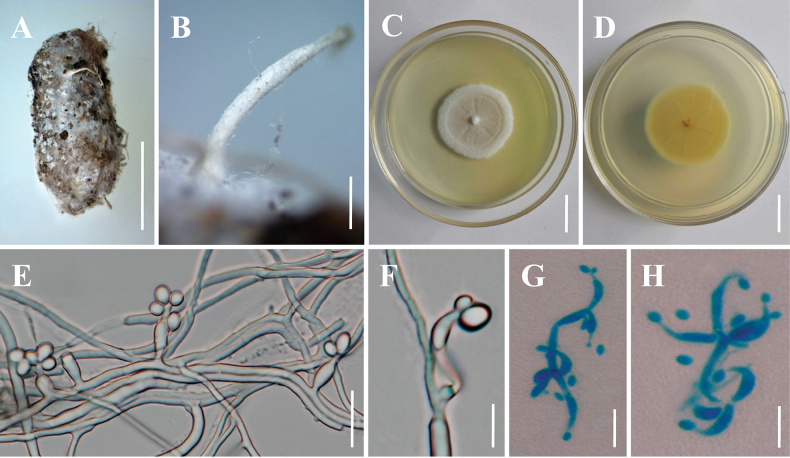
Morphology of *Amphichordaguizhouensis***A***A.guizhouensis* on bird feces **B** Synnemata **C, D** Colony obverse and reverse on PDA medium **E–H** Conidiophores, conidiogenous cells and conidia. Scale bars: 5 mm (**A**); 400 μm (**B**); 20 mm (**C–D**); 10 μm (**E, G, H**); 5 μm (**F**).

##### Other material examined.

China • Guizhou Province, Anshun City, Xixiu District, Liuguan Village (26.25°N, 106.22°E, 1269 m above sea level), on bird feces, 12 July 2023, Yao Wang (paratype: GMB 3006); ex-paratype culture, GMBC 3006).

##### Substrate.

Animal feces.

##### Distribution.

At present, known only in Anshun City, Guizhou Province, China.

##### Notes.

Phylogenetic analyses placed *A.guizhouensis* within the *Amphichorda* clade, forming a sister lineage to *A.felina* and *A.yunnanensis* with strong statistical support (BS/BS/PP = 97%/100%/1; Fig. 1). The species formed a distinct monophyletic group comprising two sampled strains, demonstrating significant genetic divergence from *A.felina* and *A.yunnanensis*. Morphologically, our observations unearthed distinct disparities among the three species. Specifically, *A.felina* exhibited phialides that were consistently flask-shaped, while *A.guizhouensis* featured phialides that were either cylindrical or flask-shaped. In contrast, *A.yunnanensis* possessed phialides ranging from ampulliform to flask-shaped. A particularly notable characteristic of *A.guizhouensis* was its relatively elongated phialides (6.0–20.8 × 1.8–3.7 µm). This unique morphological trait served as a crucial diagnostic feature, enabling clear differentiation of *A.guizhouensis* from other species within the *Amphichorda* genus (see Table 2).

**Table 2.** Hosts/substrates and asexual morphology of *Amphichorda*.

Species	Host/Substrate	Conidiophores	Phialides (μm)	Conidia (μm)	References
*Amphichordacavernicola*	Bird feces; soil; plant debris; animal feces; bat guano	Cylindrical, straight or slightly curved, occasionally branched	Fusiform or ellipsoidal, straight or irregularly bent, 4.5–8.0 × 2.0–3.0	Broadly ellipsoidal to subglobose, 2.5–4.0 × 2.0–3.5	Zhang et al. 2020
*Amphichordacoprophila*	Chipmunk, rabbit and porcupine dung	Straight or flexuous, unbranched, bearing lateral or terminal conidiogenous cells, arranged singly or in whorls	Flask-shaped, usually with a strongly bent neck, 6–10 × 2–2.5	Subglobose to somewhat ellipsoidal, 3.5–5.5 × 2–3	Guerra-Mateo et al. 2023
*Amphichordaexcrementa*	Animal feces	Cylindrical, straight or slightly curved, occasionally branched	Occasionally solitary, mostly in whorls of 2–3, basal portion cylindrical or flask-shaped, usually curved, 4.1–13.9 × 1.3–2.1	Globose to elliptical 1.7–3.0 × 1.2–2.5	Wang et al. 2024
*Amphichordafelina*	Pupae of *Anaitisefformata*; rabbit dung; moudy leaves; porcupine dung; cat dung	Straight	Solitarily or in small groups, consisting of a swollen, flask-shaped or curved, occasionally elongate basal part, 1.5–8.5 × 1.8–2.9	Subglobose, ellipsoidal or ovoidal, sometimes with a pointed base, 2.5–4.7 × 2–3.5	Wang et al. 2024
*Amphichordaguana*	Bat guano	Straight or slightly curved	Fusiform or ellipsoidal, straight or irregularly bent, 7–10 × 2–3	Broadly ellipsoid to subglobose, 4.5–5.5 × 3.5–5	Zhang et al. 2017
***Amphichordaguizhouensis***	**Animal feces**	**Cylindrical, straight or slightly curved, occasionally branched**	**Basal portion cylindrical or flask-shaped, erect or irregularly curved, tapering abruptly towards the apex, 6.0–20.8 × 1.8–3.7**	**Subglobose to ellipsoidal, 2.6–4.0 × 1.8–2.6**	**In this study**
*Amphichordakunmingensis*	Animal feces	-	Solitary, occasionally in simple whorls, basal portion cylindrical or fusiform, straight or irregularly bent, 6.1–17.5 × 1.4–2.9	Globose to elliptical 2.3–4.2 × 1.6–3.0	Wang et al. 2024
*Amphichordalittoralis*	Sediments; fragment of floating rubber tire	Straight or flexuous, commonly unbranched, bearing lateral or terminal conidiogenous cells, arranged singly or in whorls of 2–4	Flask-shaped, usually with a strongly bent neck, 6–10 (–11.5) × 1.5–2	Subglobose, 3–4 × 2.5–3	Guerra-Mateo et al. 2023
*Amphichordamonjolensis*	On PDA plate consumed by an insect	Cylindrical, bearing one or more conidiogenous cells, straight or slightly bent, solitary or synnematous, sometimes branched	Flask-shaped, straight or irregularly bent, 3.1–6.1 × 2.7–5.1	Holoblastic, 2.8–3.7 × 1.8–2.9	Leão et al. 2024
*Amphichordayunnanensis*	Wing surfaces of *Rhinolophus*	Cylindrical, straight or slightly curved, branched	Monoblastic to polyblastic, ampulliform to flask-shaped, 4–12 × 1–4	Globose to oval, slightly ellipsoid, 2–5 × 2–4	Liu et al. 2023

#### ﻿ Ovicillium pseudoattenuatum

Taxon classificationFungiHypocrealesBionectriaceae

Y. Wang & D.X. Tang sp. nov.

9AC57BDD-09D7-510A-B119-07F837A30B0A

857726

Fig. 3

##### Etymology:

“*Pseudoattenuatum*” refers to morphologically resembling *Ovicillium attenuatum*, but phylogenetically distinct.

##### Type.

Laos • Vientiane City, Mekong Riverside Park (17.96°N, 102.60°E, 674 m above sea level), from soil on the forest floor, 11 August 2024, Yao Wang (holotype as dried culture GMB 3007); ex-type culture GMBC 3007.

##### Description.

**Sexual morph**: Undetermined. **Asexual morph**: Colonies on PDA reaching 23–25 mm in diameter in 7 days at 25 °C, white to pinkish; reverse yellowish. ***Hyphae*** branched, smooth-walled, septate, hyaline, 1.2–2.8 μm wide. ***Conidiophores*** hyaline, smooth-walled, with single phialide or whorls of 2–5 phialides or verticillium-like directly from hyphae, up to 500 μm long. Phialides terminal or lateral, straight, somewhat inflated base, attenuated from the middle, sometimes undulated near the tip, 16.0–37.5 × 1.5–2.4 (X̄ = 26.8 × 2.0, n = 50) μm. ***Conidia*** smooth-walled, hyaline, ellipsoidal to cylindrical, 3.2–4.0 × 1.7–3.2 (X̄ = 3.7 × 2.3, n = 50) μm, aggregated in large globose to subglobose heads. Crystals absent. ***Chlamydospores*** absent.

**Figure 3.**
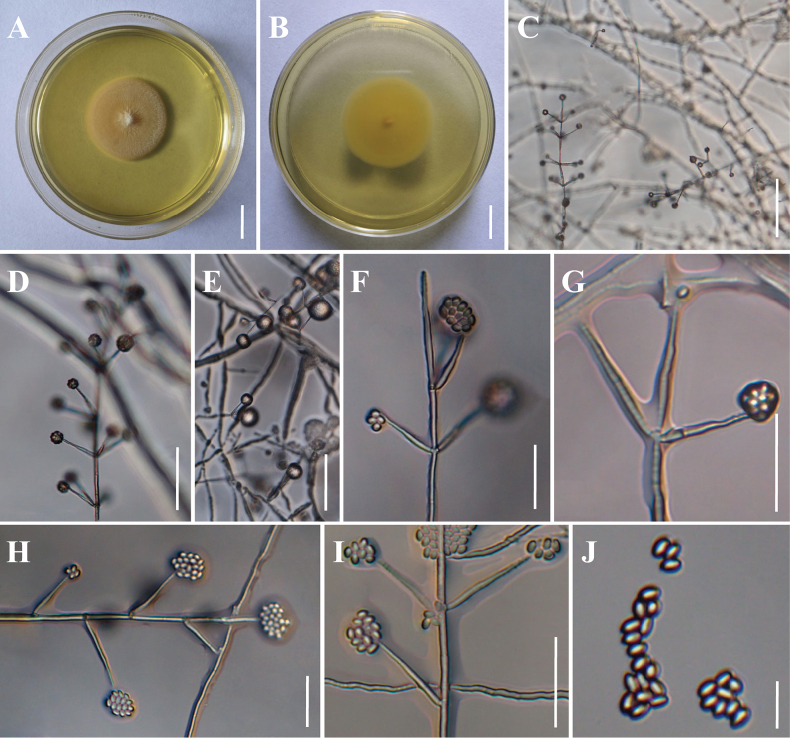
Morphology of *Ovicilliumpseudoattenuatum***A, B** Colony obverse and reverse on PDA medium **C–I** Conidiophores, phialides and conidia **J** Conidia. Scale bars: 10 mm (**A–B**); 100 μm (**C**); 50 μm (**D–E**); 25 μm (**F–I**); 10 μm (**J**).

##### Other material examined.

Laos • Oudomxay Province, Muang Xay District, Nam Kat Yorla Pa Resort (20.71°N, 102.11°E, 708 m above sea level), from soil on the forest floor, 14 August 2024, Yao Wang (living culture GMBC 3008).

##### Substrate.

Soil.

##### Distribution.

Laos.

##### Notes.

*Ovicilliumpseudoattenuatum*, isolated from forest floor soil, forms a distinct phylogenetic lineage within the *Ovicillium* genus. Multilocus phylogenetic analyses reveal its close relationship with *O.attenuatum* and *O.sinense*, supported by strong statistical values (BS_IQ_/BS_RAx_/PP = 82%/79%/0.93). Morphologically, while sharing the characteristic undulated phialide tips with *O.attenuatum*, *O.pseudoattenuatum* differs significantly in microscopic dimensions: it possesses smaller phialides (16.0–37.5 × 1.5–2.4 μm vs 25–50 × 1.7–3.3 μm) and more compact conidia (3.2–4.0 × 1.7–3.2 μm vs 3.5–5 × 2.5–3.8 μm). Distinct from *O.sinense*, which exhibits even smaller reproductive structures (phialides 16.2–25.8 × 1.7–2.4 μm; conidia 2.1–2.9 × 1.1–1.7 μm), *O.pseudoattenuatum* is further characterized by its unique ellipsoidal to cylindrical conidial morphology, a diagnostic feature distinguishing it from all known *Ovicillium* species (Table 3).

**Table 3.** Hosts/substrates and asexual morphology of *Ovicillium*.

Species	Host/Substrate	Conidiophores (μm)	Phialides (μm)	Conidia (μm)	Crystals	Chlamydospores (μm)	References
*Ovicilliumasperulatum* (Synonym: *O.napiforme*)	Soil; wood of *Sorbusaria*	Simple or mostly branched, bearing whorls of 2–4 phialides, up to 105 long, with cell walls usually thicker than those of the vegetative hyphae	Straight or slightly bent, acicular, 28–68 long, 1–2 wide at the base, with minute collarette and distinct periclinal thickening at the apex	Globose, 3–4(–5) diameter, chromophilic	Absent	Abundant, subglobose or oval, 5–10 × 5–9	Giraldo et al. 2012; Zare and Gams 2016
*Ovicilliumattenuatum*	*Auricularia* sp.	Erect, 150–500 tall, with cyanophilic encrustation, usually with whorls, but sometimes also solitary phialides	Aculeate, attenuated from the middle, mostly undulated near the tip, rather cyanophilic, measuring 25–50 × 1.7–3.3	Oval to subglobose, strongly cyanophilic, measuring 3.5–5 × 2.5–3.8, aggregated in large globose to subglobose heads	Absent	Absent	Zare and Gams 2016
*Ovicilliumoosporum*	*Theobromagileri*	Erect, solitary and verticillate, with slightly pigmented base producing solitary or verticillate phialides of up to five per node	Verticillate, measuring 20–50 × 1.2–2.2	Subglobose, oval to broadly oval, in some strains with a basal protrusion, cyanophilic, measuring 4–6 × 2.5–4	Absent	Present or absent	Zare and Gams 2016
***Ovicilliumpseudoattenuatum***	**Soil**	**Single phialide or whorls of 2–5 phialides or verticillium-like directly from hyphae, up to 500 long**	**Straight, somewhat inflated base, attenuated from the middle, sometimes undulated near the tip, 16.0–37.5 × 1.5–2.4**	**Ellipsoidal to cylindrical, 3.2–4.0 × 1.7–3.2, aggregated in large globose to subglobose heads**	**Absent**	**Absent**	**In this study**
*Ovicilliumsinense*	Pupa (Lepidoptera)	Single phialide or whorls of 2–5 phialides or verticillium-like, 17.0–21.7 × 2.3–3.0	Cylindrical, somewhat inflated base, 16.2–25.8 × 1.7–2.4, tapering to a thin neck	Globose to ovoid, 2.1–2.9 × 1.1–1.7, aggregated in large globose to subglobose heads	–	–	Chen et al. 2024
*Ovicilliumsubglobosum*	Soil	Erect, solitary and verticillate with up to four phialides per node	Measuring 25–55 × 1.5–2.2, producing conidia in large globose heads.	Subglobose with an inconspicuous protrusion at the base, rather cyanophilic, measuring 3.5–5.5 × 3.5–4.5	Absent	Absent	Zare and Gams 2016
*Ovicilliumvariecolor*	Soil	Erect, mostly branched, bearing whorls of 2–5 phialides, up to 290 long, with walls usually thicker than those of the vegetative hyphae	Straight, acicular, 18–95 long, 1–2 wide at the base, with periclinal thickening at the apex, collarette inconspicuous; some phialidic conidiogenous cells without a basal septum (adelophialides)	Subglobose or ovoid, 3–4(–5) × 2–4, slightly apiculate base, chromophilic, arranged in slimy heads	–	Absent	Giraldo et al. 2012

## ﻿Discussion

*Amphichorda* species exhibit exceptional ecological plasticity, colonizing diverse substrates including caves, marine sediments, soil, animal waste, and even biotic surfaces such as the wings of bats (*Rhinolophus* spp.). Although their primary niche is coprophilous (dung-associated)—evidenced by the recent discovery of *A.guizhouensis* and most congeners in animal excrement—their adaptability is further exemplified by colonization of specialized microhabitats. For instance, *A.felina* has been isolated from decomposing leaves and *Anaitisefformata* pupae (de Hoog 1972; Wang et al. 2024), while *A.yunnanensis* inhabits bat wing surfaces (Liu et al. 2023). Notably, three *Hapsidospora* species (*H.globosa*, *H.inversa*, and *H.stercoraria*) also exhibit obligate coprophily. This parallel ecological specialization between *Amphichorda* and *Hapsidospora* underscores their phylogenetic affinity, suggesting either a shared ancestral adaptation to fecal substrates or convergent niche evolution within the Bionectriaceae family.

The broad ecological range of *Amphichorda* is accompanied by subtle morphological distinctions, complicating taxonomic differentiation among cryptic species. Consequently, molecular data play a pivotal role in delineating species boundaries. Recent studies have employed multi-locus sequence data (ITS, nrSSU, nrLSU, *tef1α*, *rpb1*, and *rpb2*) to resolve phylogenetic relationships within the genus (Zhang et al. 2017, 2020; Guerra-Mateo et al. 2023; Liu et al. 2023; Leão et al. 2024; Wang et al. 2024). However, GenBank records (https://www.ncbi.nlm.nih.gov, accessed 20 February 2025) remain incomplete for nrSSU, *rpb1*, and *rpb2* in this group. To address this gap, we generated comprehensive molecular datasets from five distinct *Amphichorda* species. These data enrich existing genomic resources and enhance the resolution of phylogenetic reconstructions, providing deeper insights into the evolutionary history of the genus.

Our multi-locus phylogeny (nrSSU-ITS-nrLSU-*tef1α*-*rpb1*-*rpb2*) revealed a close genetic relationship among *Amphichorda*, *Hapsidospora*, *Ovicillium*, *Proxiovicillium*, and *Bulbithecium*. Consistent with Leão et al. (2024), *Amphichorda* and *Hapsidospora* form a well-supported sister clade within Bionectriaceae (BS_IQ_/BS_RAx_/PP = 93%/99%/1; Fig. 1). Additionally, *Ovicillium* clustered with *Proxiovicillium* + *Bulbithecium* as a distinct lineage, while maintaining a close genetic affinity with *Amphichorda*. Although earlier studies positioned *Ovicillium* as sister to *Proxiovicillium* (Hou et al. 2023; Leão et al. 2024), our expanded sampling of seven *Ovicillium* species [compared to two in Hou et al. (2023) and three in Leão et al. (2024)] yielded more stable topological relationships among these genera. Morphological traits further corroborate these genetic linkages: *Ovicillium* species produce large globose conidial heads, *Proxiovicillium* forms conidia in elongated chains, and *Bulbithecium* exhibits slimy conidial heads arranged in chains.

The ongoing discovery of new species in biodiversity studies is essential to our comprehension of the complexity of ecosystems and the development of life. The discovery of novel species remains pivotal for elucidating ecosystem complexity and evolutionary trajectories. Here, we propose two new species—*A.guizhouensis* (isolated from animal feces) and *O.pseudoattenuatum* (from soil)—based on integrated morphological and phylogenetic evidence. A comparative analysis of morphological traits across all *Amphichorda* and *Ovicillium* members was conducted to refine taxonomic boundaries. This study not only clarifies evolutionary relationships within the two genera but also advances the systematic understanding of biodiversity in Bionectriaceae.

## ﻿Acknowledgments

In this section, you can acknowledge any support given which is not covered by the author contribution or funding sections. This may include administrative and technical support, or donations in kind (e.g., materials used for experiments).

## Supplementary Material

XML Treatment for Amphichorda guizhouensis

XML Treatment for Ovicillium pseudoattenuatum

## ﻿Additional information

### Author ORCIDs

Yao Wang https://orcid.org/0000-0002-1262-6700

Hui Chen https://orcid.org/0009-0008-0291-3571

AuthorName https://orcid.org/0000-0002-4333-9106

### Data availability

All of the data that support the findings of this study are available in the main text.
